# Absence of association between *Plasmodium falciparum* small sub-unit ribosomal RNA gene mutations and in vitro decreased susceptibility to doxycycline

**DOI:** 10.1186/s12936-015-0878-x

**Published:** 2015-09-17

**Authors:** Tiphaine Gaillard, Nathalie Wurtz, Sandrine Houzé, Kanlaya Sriprawat, Chirapat Wangsing, Véronique Hubert, Jacques Lebras, François Nosten, Sébastien Briolant, Bruno Pradines, The French National Reference Centre for Imported Malaria Study Group

**Affiliations:** Unité de Parasitologie, Département d’Infectiologie de Terrain, Institut de Recherche Biomédicale des Armées, Marseille, France; Unité de Recherche sur les Maladies Infectieuses et Tropicales Emergentes, Aix Marseille Université, UM 63, CNRS 7278, IRD 198, Inserm 1095, Marseille, France; Fédération des Laboratoires, Hôpital d’Instruction des Armées Saint Anne, Toulon, France; Centre National de Référence du Paludisme, Marseille, France; Laboratoire de Parasitologie-Mycologie, Centre National de Référence du Paludisme, APHP, Hôpital Bichat-Claude Bernard, Paris, France; IRD UMR216, Mère et enfant face aux infections tropicales, Paris, France; PRES Sorbonne Paris Cité, Faculté des Sciences Pharmaceutiques et Biologiques, Université Paris Descartes, Paris, France; Shoklo Malaria Research Unit, Mahidol-Oxford Tropical Medicine Research Unit, Faculty of Tropical Medicine, Mahidol University, Mae Sod, Thailand; Centre for Tropical Medicine, University of Oxford, Oxford, UK; Direction Inter-Armées du Service de Santé, Cayenne, French Guiana; Laboratoire de Parasitologie, Institut Pasteur de la Guyane, Cayenne, French Guiana; Unité de Parasitologie et d’Entomologie, Département des Maladies Infectieuses, Institut de Recherche Biomédicale des Armées, Brétigny sur Orge, France

**Keywords:** Malaria, *Plasmodium falciparum*, In vitro, Anti-malarial, Molecular marker, Doxycycline, Small ribosomal sub-unit RNA gene, *pfssrRNA*, 16S rRNA

## Abstract

**Background:**

Doxycycline is an antibiotic used in combination with quinine or artesunate for malaria treatment or alone for malaria chemoprophylaxis. Recently, one prophylactic failure has been reported, and several studies have highlighted in vitro doxycycline decreased susceptibility in *Plasmodium falciparum* isolates from different areas. The genetic markers that contribute to detecting and monitoring the susceptibility of *P. falciparum* to doxycycline, the *pfmdt* and *pftetQ* genes, have recently been identified. However, these markers are not sufficient to explain in vitro decreased susceptibility of *P. falciparum* to doxycycline. In this paper, the association between polymorphism of the small sub-unit ribosomal RNA apicoplastic gene *pfssrRNA* (PFC10_API0057) and in vitro susceptibilities of *P. falciparum* isolates to doxycycline were investigated.

**Methods:**

Doxycycline IC_50_ determinations using the hypoxanthine uptake inhibition assay were performed on 178 African and Thai *P. falciparum* isolates. The polymorphism of *pfssrRNA* was investigated in these samples by standard PCR followed by sequencing.

**Results:**

No point mutations were found in *pfssrRNA* in the Thai or African isolates, regardless of the determined IC_50_ values.

**Conclusions:**

The *pfssrRNA* gene is not associated with in vitro decreased susceptibility of *P. falciparum* to doxycycline. Identifying new in vitro molecular markers associated with reduced susceptibility is needed, to survey the emergence of doxycycline resistance.

## Background

Doxycycline is an effective anti-malarial prophylactic drug when administered as a monotherapy 1 day before, daily during, and for 4 weeks after return from travel to an area where malaria is endemic. Doxycycline is currently a recommended chemoprophylactic regimen for travellers visiting areas where malaria is endemic and has a high prevalence of chloroquine or multidrug resistance [1–3]. The World Health Organization also recommends doxycycline in combination with quinine or artesunate as the second-line treatment for uncomplicated *Plasmodium falciparum* malaria [2].

Most prophylactic failures of doxycycline against *P. falciparum* were associated with the use of inadequate, low doses or poor compliance [4–6]. However, resistance could also explain prophylactic failures with doxycycline. Cyclines resistance has been documented in *Plasmodium berghei* as a consequence of minocycline drug pressure in a *P.berghei* murine malaria model [7]. Recently, one prophylactic failure has been reported [8].

A Bayesian mixture modelling approach identified three different phenotypes (low, medium, and high doxycycline IC_50_ phenotypic groups) among *P. falciparum* African clinical isolates [9, 10]. Using 90 isolates from 14 African countries, it was demonstrated that increases in copy numbers of *P. falciparum* metabolite drug transporter gene (*Pfmdt*, PFE0825w) and *P. falciparum* GTPase TetQ gene (*PfTetQ*, PFL1710c) are associated with reduced susceptibility to doxycycline [11], and this association was later confirmed in African *P. falciparum* isolates [9]. In addition, isolates with PfTetQ KYNNNN motif repeats <3 are associated with in vitro reduced susceptibility to doxycycline and with a significantly higher probability of having an IC_50_ above the doxycycline resistance threshold of 35 µM (odds ratio of 15) [11, 12]. The isolate obtained from the patient with prophylactic resistance to doxycycline harboured two copies of *pfmdt* and two PfTetQ KYNNNN motif repeats [8], consistent with previous in vitro data [12].

However, some recent publications have demonstrated that these molecular markers were certainly not only encountered in cases of reduced susceptibility to doxycycline [13, 14] and were not associated with resistance in Thai isolates [14]. Therefore, it is necessary to investigate other hypotheses. Based on bacterial world, proteins homologue to those implicated in doxycycline resistance in bacteria were identified in silico in *P. falciparum*.

Indeed, cyclines bind to proteins S4, S7, S9, and S17 of the 30S small ribosomal sub-unit and various ribonucleic acids of the 16S ribosomal RNA, preventing the binding of aminoacyl-transfer RNA to site A of the ribosome and thus blocking the elongation step of translation in bacteria [15]. Specific mutations in genes coding these targets can confer resistance to tetracyclines in bacteria. However, no point mutation was found in small sub-unit plastid ribosomal homologue plasmodial genes in African isolates (*pfrps7*, *pfrps9*, and *pfrps17*, although S7, S9, and S17) [11]. It has been also shown that resistance to tetracycline was mediated by mutations in the 16S rRNA gene, particularly in *Helicobacter pylori* or in *Propionibacterium acnes* [16–18]. An analogue of this gene exists in *P. falciparum* apicoplast, the small sub-unit ribosomal RNA gene, the *pfssrRNA* gene, (PFC10_API0057) [19–22]. First, the *pfssrRNA* gene shares 58 and 62 % identities with the 16S rRNA gene of *Propionibacterium acnes* and *Helicobacter pylori*, respectively. Secondly, this gene belongs to the apicoplast, an organelle related to the chloroplast of plant cells that contains its own genome-encoding, prokaryote-like, ribosomal RNAs, tRNAs and some proteins [23]. Three studies confirmed the specific action of cyclines on the apicoplast of *P. falciparum* [24–26]. A parasite exposed to 1 µM of doxycycline for 20 h presented during the next cycle (72 h), the inhibition of apicoplastic replication visualized by confocal fluorescence microscopy, electron microscopy and an analysis of the parasite transcriptome [24]. The most recently published study confirms the action of doxycycline on the apicoplast but in two stages, with an immediate toxic effect and a toxic effect measurable after cell division [25]. A proteomic approach confirmed the specific deregulation of proteins involved in apicoplast metabolism after doxycycline treatment [27].

Thus, the aim of this study was to identify specific point mutations in this plasmodial ribosomal gene, according to what is observed in other species, to determine whether this gene could be involved in reduced susceptibility to doxycycline. For this purpose, the apicoplastic *pfssrRNA* gene from the 89 African and 89 Thai *P. falciparum* isolates, belonging to phenotypic groups differing in doxycycline IC_50_ values and already analysed for *pftetQ* and *pfmdt* genes, was sequenced and analysed [9, 14].

## Methods

### *Plasmodium falciparum* isolates

A total of 89 African *P. falciparum* isolates, obtained at the French National Reference Centre for Imported Malaria, Hôpital Bichat, Paris, from patients hospitalized with malaria after having returned to France between January 2006 and December 2010, and 89 isolates obtained from the Shoklo Malaria Research Unit (Mae Sot, Thailand) from patients infected with *P. falciparum* from 2001 to 2010, were used. These isolates were previously tested to evaluate their *pfmdt* and *pftetQ* genes copy numbers [9, 14].

### Consent

Informed consent was not required as the sampling procedures and testing are part of the French national recommendations for the care and surveillance of malaria.

Concerning the Thai isolates, written informed consent translated into the patient’s own language was obtained from each participant, whose signature was witnessed. The studies were approved by the Ethics Committees of the Faculty of Tropical Medicine, Mahidol University and Oxford University.

### Amplification and sequencing of *pfssrRNA* gene

*PfssrRNA* (PFC10_API0057) was amplified by polymerase chain reaction (PCR) using the following primers: 5′-AGCTAATGGTGAGATTTGAACTCA-3′ (forward) and 5′-CGTCGTGAGACAGTTCGGTC-3′ (reverse) (Eurogentec, Angers, France), designed with the NCBI/Primer-BLAST online tool.

The reaction mixture included 2 µl of genomic DNA, 2.5 µl of 10× reaction buffer (Eurogentec), 0.5 µM of each primer, 200 µM of deoxynucleoside triphosphate mixture (dGTP, dATP, dTTP and dCTP) (Euromedex, Souffelweyersheim, France), and 1.5 mM of MgCl_2_ and 1.25 units of RedGoldStar^®^ DNA polymerase (Eurogentec) in a final volume of 25 µL. The thermal cycler (T3 Biometra, Archamps, France) was programmed as follows: an initial 94 °C for 2 min followed by 40 cycles of 94 °C for 30 s, 55 °C for 30 s and 60 °C for 2 min, and a final extension step of 60 °C for 5 min. The PCR products were loaded on 1 % agarose gel containing 0.5 μg/mL ethidium bromide. Amplicons were purified using the QIAquick 96 PCR BioRobot Kit and an automated protocol on the BioRobot 8000 workstation (Qiagen, Courtaboeuf, France). The purified fragments were sequenced using BigDye Terminator v3.1 Cycle Sequencing Kit (Applied Biosystems) using the following primers: 5′-ACTAGTGTATTTCGGTTAACAGCCG-3′ (forward), 5′-ACCCTTATCAAGAGTATGTTTTAACCAT-3′ (reverse) and Pf_SSU_rRNA_R1481 CTTAAGAACTTATTCACCGCTA (reverse). The sequence reaction products were purified using the BigDye XTerminator^®^ Purification Kit (Applied Biosystems), in accordance with the manufacturer’s instructions. The purified products were sequenced using an ABI Prism 3100 analyser (Applied Biosystems), and the sequences were analysed using Vector NTI advance (TM) software (version 11, Invitrogen, Cergy Pontoise, France).

## Results

In *Helicobacter pylori*, tetracycline resistance has not been associated with efflux or ribosomal protection proteins but rather attributed to mutations in the 16S rRNA-encoding genes that affect the binding site of tetracycline [16–18]. Tetracycline resistance mediated by mutations in the 16S rRNA was first found in *Propionibacterium acnes*, and a mutation from G to C was reported at position 1058 (*Escherichia coli* numbering) in their 16S rRNA genes [17]. A triplet mutation in the same 16S rRNA domain (965–967; *E. coli* numbering) was also found [24, 28–30] and is located in the primary tetracycline-binding site [1, 15]. However, the sequencing of *pfssrRNA* did not reveal a polymorphism in *P. falciparum*. There was no single nucleotide polymorphism in the *pfssrRNA* gene in either the 89 African isolates, regardless of the phenotypic group for doxycycline (group A of low doxycycline IC_50_ [mean IC_50_ = 3.88 µM; confident interval 95 % (CI 95 %) [3.39–4.37], no = 30], group B of moderate IC_50_ [mean IC_50_ = 16.97 µM; CI 95 % [16.45–17.49]; no = 30]) and group C of high IC_50_ [mean IC_50_ = 34.60 µM, CI 95 % [31.3–37.9], no = 29), or the 89 Thai isolates (group A [mean IC_50_ = 3.64 µM, CI 95 % [3.29–3.99], no = 30], group B [mean IC_50_ = 14.73 µM, CI 95 % [14.6–14.85], no = 30] and group C [mean IC50 = 28.94 µM, CI 95 % [26.51–31.37], no = 29]). No sequence polymorphism in the *pfssrRNA* gene was observed by comparison with the reference strain 3D7. This gene was not associated with reduced susceptibility to doxycycline in either African or Thai *P. falciparum* isolates and the small sub-unit ribosomal RNA seemed to be not a target for doxycycline.

## Conclusions

The decreased susceptibility of *P. falciparum* to doxycycline is certainly multigenic. *Pfmdt* and *pftetQ* genes polymorphism and number of copies are involved partly to the decreased susceptibility. Intensive research into identifying in vitro markers associated with decreased susceptibility should allow survey of the emergence of doxycycline resistance. Another hypothesis to be explored is some apicoplastic genes, which could be involved in artemisinin resistance [31], such as *arps10*, encoding the apicoplast ribosomal protein S10 precursor, and *fd*, encoding the ferredoxin protein, a key component of the apicoplast electron transport chain.
